# Supplementary material for: Probing the Role of the Hinge Segment of Cytochrome P450 Oxidoreductase in the Interaction with Cytochrome P450
Source: Int J Mol Sci. 2018 Dec 6;19(12):3914. doi: 10.3390/ijms19123914 (PMC6321550; doi:10.3390/ijms19123914)
Supplement: Supplementary file 1 [file ijms-19-03914-s001.pdf]

Supplemental Material

PROBING THE ROLE OF THE HINGE SEGMENT OF CYTOCHROME P450 OXIDOREDUCTASE IN THE INTERACTION WITH CYTOCHROME P450

Diana Campelo[1], Francisco Esteves[1], Bernardo Brito Palma[1], Bruno Costa Gomes[1], José Rueff[1], Thomas Lautier[2], Philippe Urban[2], Gilles Truan[2] and Michel Kranendonk[1*]

[1] Center for Toxicogenomics and Human Health, Genetics, Oncology and Human Toxicology, NOVA Medical School, Faculdade de Ciências Médicas, Universidade Nova de Lisboa, Lisbon, Portugal.

[2] LISBP, Université de Toulouse, CNRS, INRA, INSA, Toulouse, France.

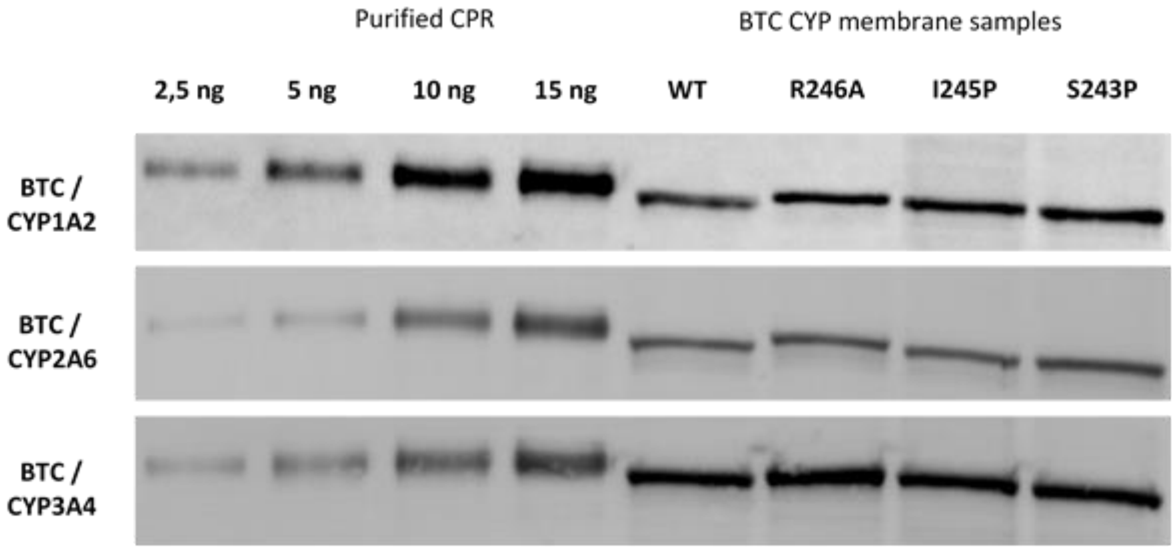

Supplemental Figure S1: Immuno-detection of human CPR variants in membrane preparations.

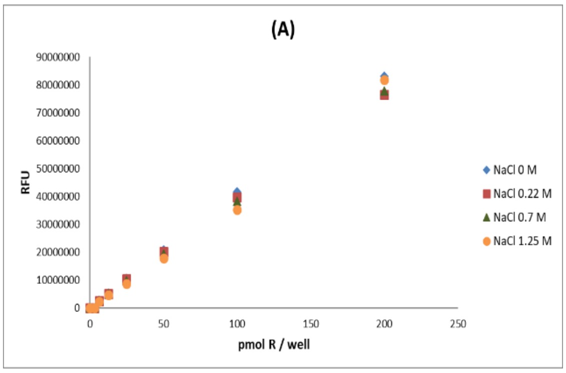

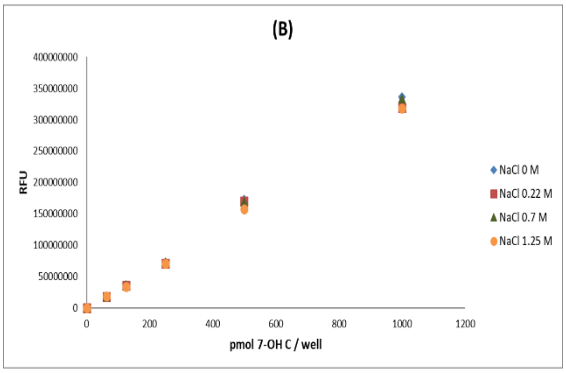

(**a**) (**b**)

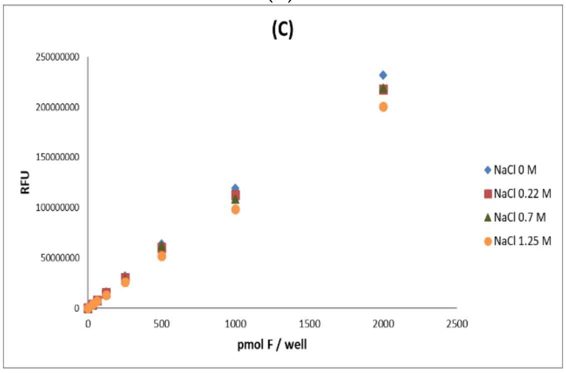

(**c**)

Supplemental Figure S2: Fluorescence of resorufin (R), 7-OH coumarin (7-OH C) and fluorescein (F) with increasing NaCl concentrations, in panel A, B and C, respectively.
